# P-2148. Prospective Study on Microbiological Profile of Fungal Isolates and its Antibiogram in a Tertiary care hospital in South India

**DOI:** 10.1093/ofid/ofae631.2302

**Published:** 2025-01-29

**Authors:** Vaishnavi Velmani, Sriram Radhakrishnan, Leena Muppa, Sureshkumar Dorairajan

**Affiliations:** C.L.Baid Metha College of Pharmacy, Affiliated to the Tamil Nadu Dr. MGR Medical University, Chennai, Tamil Nadu, India; C.L.Baid Metha College of Pharmacy, Affiliated to the Tamil Nadu Dr. MGR Medical University, Chennai, Tamil Nadu, India; C.L.Baid Metha College of Pharmacy, Affiliated to The Tamil Nadu Dr. MGR Medical University, Chennai, Tamil Nadu, India; Best of IDs, Chennai, Tamil Nadu, India

## Abstract

**Background:**

Fungal infections are increasingly recognized as significant causes of morbidity and mortality, particularly in immunocompromised individuals. Despite their importance, there is limited data on the microbiological profile and antimicrobial resistance patterns of fungal isolates in South India. This prospective study aims to fill this gap by characterizing fungal isolates and determining their antibiograms in a tertiary care hospital, providing crucial insights for clinical management and infection control strategies.

**Figure:**
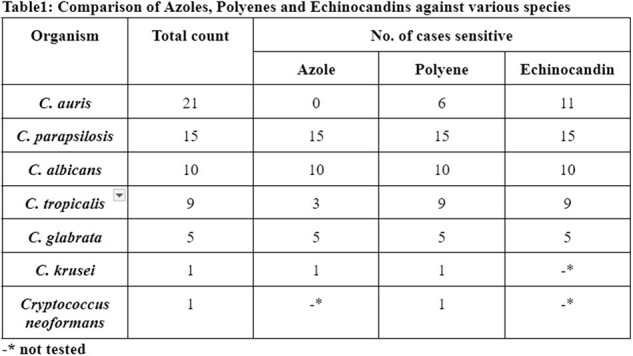

**Methods:**

A Prospective Observational Study was done under the guidance of an Infectious diseases Specialist by collecting the culture positive fungal isolates from the Microbiology department for the 1 year duration of April 2023 to March 2024. Data including patient age, gender, specimen, type of fungus isolated, its sensitivity pattern, average time required for identification of the species and requirement of ICU admission were analyzed. The three classes of antifungals including polyenes, azoles and echinocandins were included. Data was entered and analyzed using Microsoft excel.

**Results:**

A total of n=62 fungal isolates were isolated. The average age of the patients was 49 years with a higher prevalence in male (n=42) compared to females (n=20). Fungal species have been isolated more commonly from blood samples followed by urine samples, tissues, body fluids. In all the cases tested, Azoles, Polyenes and Echinocandins were susceptible in 57%, 76% and 80% of the cases respectively. Average time taken for identification of the subspecies was 124 hrs (∼ 5 days). *Candida auris* was the most common isolate found, which developed complete resistance to azoles, partially to polyenes, while echinocandin has been found to be a reliable drug of choice. The spectrum and efficacy of Echinocandins were higher compared to azoles and polyenes.

**Conclusion:**

*Candida auris* was found as the most resistant species and echinocandins have been found as a reliable drug of choice compared to Azoles and Polyenes antifungals. This study highlights the need for rapid diagnostic procedures for rapid identification of the species grown in the culture, thereby enabling rational antifungal therapy. As long as the reporting time delays, initiation of right antifungal therapy also delays.

**Disclosures:**

All Authors: No reported disclosures

